# Clinical Predictors of Ultrasound-Guided Cervical Medial Branch Pulsed Radiofrequency Outcomes: A Cohort Study

**DOI:** 10.3390/diagnostics16040590

**Published:** 2026-02-15

**Authors:** Ümit Akkemik, Sinan Oğuzhan Ulukaya, Mustafa Şen, Mehmet Sacit Güleç

**Affiliations:** 1 Department of Algology, Faculty of Medicine, Eskişehir Osmangazi University, 26040 Eskişehir, Turkey; sinanulukaya@outlook.com (S.O.U.); sacitg@yahoo.com (M.S.G.); 2 Department of Algology, Eskişehir City Hospital, 26080 Eskişehir, Turkey; menings@hotmail.com

**Keywords:** cervical facet joint pain, pulsed radiofrequency, ultrasound-guided, medial branch block, predictive factors

## Abstract

**Background/Objectives:** Cervical facet joints are a common source of chronic neck pain, yet factors predicting treatment response to pulsed radiofrequency remain poorly defined. This study aimed to identify predictors of treatment success following ultrasound-guided cervical medial branch pulsed radiofrequency in patients with chronic cervical facet joint pain. **Methods:** This retrospective cohort study included 54 patients with chronic cervical facet joint pain who had positive response to diagnostic block. Pain intensity and functional disability were assessed at baseline and at 1-, 3-, and 6-months post-procedure, with treatment success defined as ≥50% pain reduction at 6 months. **Results:** The success rate was 35.2%, and multivariate logistic regression identified four independent predictors: presence of paraspinal tenderness on physical examination, shorter pain duration, lower baseline pain intensity, and lower baseline disability. **Conclusions:** These findings suggest that patients with localized facet joint pathology manifesting as paraspinal tenderness, shorter symptom duration, and lower baseline severity are most likely to benefit from this intervention, supporting early referral and careful clinical selection to optimize treatment outcomes.

## 1. Introduction

Neck pain is a common and often disabling condition that contributes to considerable pain, disability, and healthcare burden worldwide. The Global Burden of Disease Study 2020 reported that neck discomfort impacted 203 million individuals worldwide, with forecasts suggesting an increase to 269 million by 2050 [1]. The age-standardized prevalence rate is significantly elevated in females (2890 per 100,000) relative to males (2000 per 100,000), with prevalence reaching its zenith between the ages of 50 and 74 for both genders. Neck pain contributes to substantial years lived with disability and represents a significant burden during the most productive working years, resulting in considerable economic and social consequences globally [1].

Cervical facet joints are identified as the primary pain source in 36–67% of patients with chronic neck pain. These delicate diarthrodial articulations contain nociceptive elements within their joint capsules that can act as independent pain generators. The facet joints are innervated by the medial branches of the dorsal ramus, which play crucial roles in both pain sensation and proprioception [2]. The confirmation of facet-mediated pain is generally achieved with an anesthetic blockade of the nerves innervating the facet joints.

Various treatment modalities have been proposed for managing chronic cervical joint pain, including radiofrequency (RF) procedures, physiotherapy, therapeutic exercise, and cervical manipulations. The American Society of Interventional Pain Physicians (ASIPP) designated both radiofrequency procedures and facet joint steroid or anesthetic injections as recommended treatment options [3]. For carefully selected patients with chronic cervical facet joint pain who have not responded to conservative measures and have positive diagnostic medial branch blocks, radiofrequency procedures represent an evidence-based therapeutic option. According to a recent systematic review evaluating interventional treatment strategies for cervicogenic headache and cervical spine disorders, PRF is associated with a better safety profile and fewer complications compared to other ablative techniques, with studies demonstrating significant long-lasting pain relief and sustained therapeutic benefits [4].

Pulsed radiofrequency (PRF) has emerged as an alternative to conventional radiofrequency (CRF) denervation for chronic pain management. While CRF creates thermal lesions (typically around 67 °C) causing irreversible tissue destruction and denaturation, PRF avoids tissue denaturation by maintaining average temperatures of 42 °C through short radiofrequency pulses (commonly 20 ms pulses at 2 Hz frequency), preserving neural architecture while providing therapeutic benefit through neuromodulatory mechanisms [5].

The evolution of image guidance for pain interventions has significantly improved procedural safety and accuracy. Traditional fluoroscopy-guided approaches involve ionizing radiation exposure and may require multiple orthogonal views to assess needle depth adequately. Ultrasound guidance has emerged as an attractive alternative, offering real-time visualization of soft tissues and elimination of radiation exposure [6]. Previous cadaveric research has established that ultrasound-guided CMB needle placement provides clinically acceptable accuracy with excellent safety outcomes [7].

While studies have examined predictors of response to interventional procedures in the lumbar spine [8,9,10], and a recent study investigated various clinical and radiological parameters, including sagittal alignment, to predict outcomes of conventional fluoroscopy-guided cervical facet PRF treatment [11], no studies in the literature have systematically analyzed the demographic, clinical, and radiological factors specifically associated with outcomes following ultrasound-guided CMB PRF treatment.

Identifying these prognostic indicators would allow physicians to formulate individualized treatment strategies, enhance patient selection, and increase the cost-effectiveness of care delivery. This retrospective study aims to identify and analyze the demographic, clinical, and radiological factors influencing treatment outcomes in patients who received ultrasound-guided CMB PRF treatment for chronic cervical facet joint pain, as well as to assess the prognostic significance of these factors. This study aims to enhance evidence-based patient selection criteria and tailored pain management strategies for chronic cervical joint pain by analyzing predictive characteristics.

## 2. Materials and Methods

### 2.1. Study Design and Setting

This retrospective cohort study was conducted at the Department of Pain Medicine, Eskişehir Osmangazi University Faculty of Medicine, Turkey. The study protocol was approved by the Institutional Review Board of Eskişehir Osmangazi University (approval date and number: 07.10.2025/32). All procedures were performed in accordance with the ethical standards of the institutional research committee and with the 1964 Helsinki Declaration and its later amendments. As a retrospective cohort study, no study-specific interventions were introduced; all data were extracted from existing clinical records of procedures performed as part of routine clinical care.

### 2.2. Study Population

Medical records of all patients who underwent ultrasound-guided CMB PRF treatment for chronic cervical joint pain between February 2022 and February 2025 were reviewed. Inclusion criteria were: (1) age ≥ 18 years; (2) chronic neck pain (duration ≥ 3 months) attributed to cervical facet joint origin; (3) positive response to a single diagnostic CMB block; (4) ultrasound-guided CMB PRF treatment; and (5) availability of minimum 6-month follow-up data. Exclusion criteria included: (1) specific cervical pathology (malignancy, infection, fracture, myelopathy); (2) history of prior cervical spine surgery; (3) other interventional procedures performed during the follow-up period; and (4) incomplete follow-up or missing data.

### 2.3. Diagnostic Medial Branch Block

All diagnostic block procedures were performed under ultrasound guidance with local anesthetic injection at the needle insertion site. Since each facet joint possesses dual innervation, the medial branches at the joint level and the one superior to it were anesthetized for each symptomatic joint. For C2–3 facet joint involvement, the third occipital nerve was targeted. Following negative aspiration, 0.5 mL of 0.5% bupivacaine was injected at each target site [12]. A positive diagnostic block was defined as ≥50% concordant pain relief during normal activities for a minimum of 4 h.

### 2.4. Ultrasound-Guided Pulsed Radiofrequency Procedure

**Figure 1:**
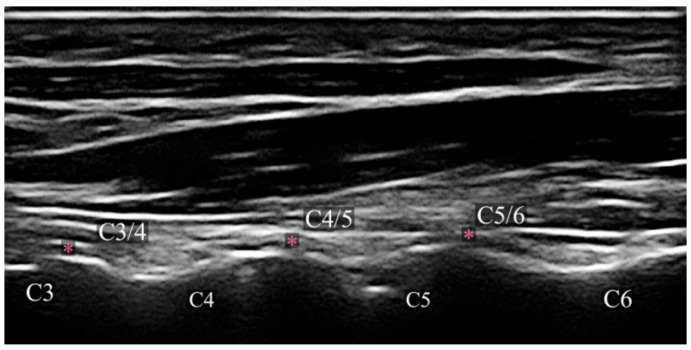
Sagittal ultrasound view of the cervical spine demonstrating the articular pillars. The articular pillars of C3, C4, C5, and C6 vertebrae are visualized as a continuous wavy hyperechoic line. The cervical medial branch target points at the waist of each articular pillar are marked with asterisks (*) at the C3/4, C4/5, and C5/6 levels.

All PRF procedures were performed by experienced interventional pain physicians using high-resolution ultrasound guidance with a 12 to 5 MHz linear transducer (MyLab™X7 Ultrasound system; Esaote S.p.A., Genova, Italy). Patients were placed in the lateral decubitus posture, with the head adequately supported by a pillow and the side of interest oriented upward. The ultrasound transducer was first positioned on the lateral side of the neck in the sagittal plane to acquire a longitudinal image of the articular pillars. A further 90° movement of the transducer yielded an axial plane scan, revealing the CMB navigating the articular pillars between the multifidus and longissimus muscles. The CMBs are visualized sonographically as curvilinear hypoechoic bands encircling the laterally curved segments of the articular pillars (Figure 1) [7].

**Figure 2:**
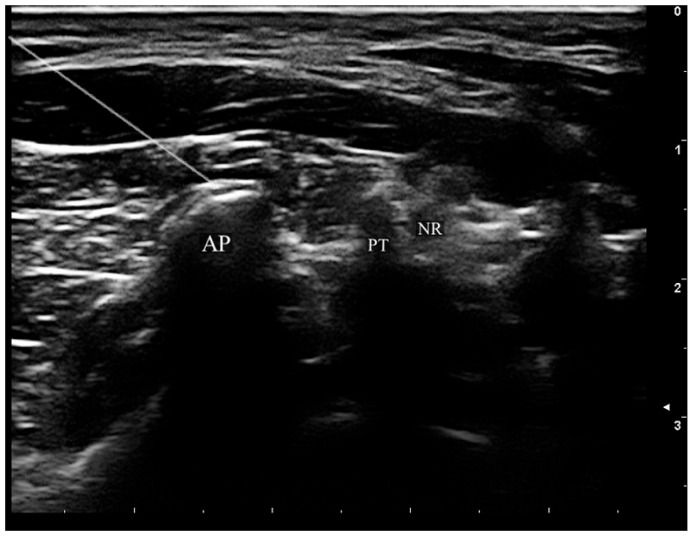
Axial ultrasound view demonstrating ultrasound-guided cervical medial branch pulsed radiofrequency needle placement. The radiofrequency needle (hyperechoic linear structure, upper left) is visualized approaching the target point at the waist of the articular pillar (AP) using an in-plane technique. PT, posterior tubercle; NR, nerve root.

Under real-time ultrasound visualization, a 22-gauge, 100-mm radiofrequency cannula with a 5-mm active tip(SMK-S1005-22; Abbott, St. Paul, MN, USA) was advanced using an in-plane technique toward the target point at the waist of the articular pillar (Figure 2). The probe is subsequently rotated to acquire a coronal image, confirming that the needle is positioned centrally within the targeted articular pillar. Further, correct needle placement was confirmed by sensory stimulation at 50 Hz, achieving concordant paresthesia at ≤0.5 V. Motor stimulation at 2 Hz was performed to exclude proximity to the ventral ramus. Following confirmation of appropriate electrode positioning, 0.5 mL of 1% lidocaine was injected through each cannula to mitigate procedural discomfort. PRF was then applied at 42 °C for 120 s using 20-ms pulses at 2 Hz frequency, with two cycles performed at each target level.

### 2.5. Data Collection

Data was collected from the hospital’s computerized medical records database. Demographic characteristics comprise age, gender, body mass index (BMI), and occupation. Occupational physical demands were classified according to the strength levels defined by the U.S. Bureau of Labor Statistics Occupational Requirements Survey (ORS): sedentary (lifting ≤ 10 lbs), light (lifting ≤ 20 lbs), medium (lifting 20–50 lbs), and heavy (lifting 50–100 lbs) [13].

Clinical variables collected included pain duration, pain localization (right, left, bilateral), presence of pain radiating to the head (cephalad radiation), shoulder radiation, history of trauma, smoking status, and comorbidities (diabetes mellitus, hypertension, depression, anxiety). Medication use was recorded, including nonsteroidal anti-inflammatory drugs (NSAIDs), opioids, and gabapentinoids. Physical examination findings documented included pain exacerbated by neck extension and/or rotation, paraspinal tenderness (assessed by firm digital pressure approximately 4 kg over the posterior cervical facet joints C2–7, considered positive when reproducing characteristic pain or eliciting pain ≥ 4/10; inter-rater reliability was not formally assessed), and segmental tenderness.

Radiological characteristics were obtained from cervical magnetic resonance imaging (MRI) and computed tomography (CT) reports. Cervical facet joint degeneration was classified according to a computed tomography-based grading system: Grade I (normal joint architecture), Grade II (initial degenerative alterations such as joint space reduction, cyst development, or minor osteophytes absent hypertrophy), Grade III (severe degeneration characterized by facet joint hypertrophy resulting from substantial osteophytes without bony fusion), and Grade IV (total bony fusion/ankylosis). Grades were classified for analysis as normal (Grade I), mild (Grade II), moderate (Grade III), and severe (Grade IV) [14]. Disc degeneration was assessed according to the Pfirrmann classification [15]. PRF procedure characteristics recorded included treatment laterality (right, left, bilateral), number of treated levels, and specific segmental levels treated.

### 2.6. Outcome Measures

Outcome data was retrieved from the hospital electronic medical records. The primary measure was pain intensity assessed using the Visual Analog Scale (VAS, 0–10). Functional disability was evaluated using the validated Turkish version of the Neck Disability Index (NDI, 0–50) [16]. VAS and NDI scores at baseline and at 1-, 3-, and 6-month post-procedure were extracted from routine clinical follow-up records.

Treatment success was defined as ≥50% reduction in VAS score at 6 months post-procedure, consistent with IMMPACT recommendations [17]. Medication dose changes (increased, unchanged, decreased) at 6 months were also documented.

### 2.7. Statistical Analysis

Statistical analyses were conducted utilizing Statistical Package for the Social Sciences version 26.0 (IBM Corp., Armonk, NY, USA). Categorical variables were expressed as counts and percentages [*n* (%)], whilst continuous variables were represented as median and interquartile range [median (IQR)]. The normality of distribution for continuous data was evaluated using the Kolmogorov–Smirnov test. The chi-square test or Fisher’s exact test (used when anticipated cell counts were below 5) was utilized for categorical variable comparisons across groups. The Mann–Whitney U test was utilized to compare continuous variables between two independent groups. The Friedman test was employed to assess variations in VAS and NDI scores across time. Upon detecting statistical significance, pairwise comparisons were conducted utilizing the Bonferroni-adjusted Wilcoxon signed-rank test.

To evaluate the clinically meaningful level of pain improvement, an anchor-based approach was used to calculate the minimum clinically important difference (MCID) as the mean change score of patients who demonstrated 20% to 50% VAS reduction at 6 months. Six-month treatment outcome was defined as a binary categorical variable: “successful” for patients showing ≥50% reduction in VAS score and “unsuccessful” for those below this threshold.

Univariate logistic regression studies were initially conducted to investigate factors related to treatment success. Variables with *p* < 0.05 in univariate studies were deemed potential candidates for the multivariate model. The multivariate logistic regression model was developed utilizing the Backward Wald approach, and only variables that provided significant independent contributions were included in the final model. Multicollinearity among variables was evaluated using variance inflation factors obtained from linear regression, with values below 5 being acceptable. The relationship between continuous predictors was assessed using Spearman’s correlation coefficient. The assessment of model discrimination was conducted via the area under the receiver operating characteristic curve, while calibration was examined through the Hosmer–Lemeshow goodness-of-fit test. Results were presented as odds ratios accompanied by 95% confidence intervals.

Post hoc power analysis was performed using the two-proportion comparison method based on paraspinal tenderness, which showed the strongest association with treatment success and remained as an independent predictor in multivariate analysis. The treatment success rate was 61.5% (16/26) in the paraspinal tenderness positive group and 10.7% (3/28) in the paraspinal tenderness negative group. With a two-tailed alpha level of 0.05, the power of the study to detect the observed difference between the two groups was 97% (power = 0.9743). To validate variable selection, all 256 possible subsets of the 8 univariate-significant candidates were compared using AIC and BIC. The backward Wald model ranked #3 by AIC (ΔAIC = 0.91 from the best model, indicating essentially equivalent fit), and #1 by BIC, confirming the robustness and parsimony of the final model.

## 3. Results

### 3.1. Patient Selection

A total of 65 patients who underwent ultrasound-guided PRF treatment for chronic cervical facet joint pain during the study period were evaluated. Of these patients, 4 (6.2%) were excluded due to incomplete follow-up, 3 (4.6%) due to missing data, 2 (3.1%) due to undergoing cervical disc herniation surgery, and 2 (3.1%) due to receiving a different interventional procedure during the study period. Consequently, a total of 54 patients (83.1%) who met the inclusion and exclusion criteria were enrolled in the study (Figure 1).

### 3.2. Demographic and Clinical Characteristics of Patients

The demographic, clinical, and radiological attributes of the 54 patients are delineated in Table 1. The majority were female (57.4%), with a median age of 54 years and a median BMI of 28.1 kg/m^2^.

Most patients were engaged in medium (42.6%) or heavy (25.9%) physical work. Median pain duration was 21 months, with unilateral pain predominating (77.8%). The most common comorbidities were smoking (40.7%), anxiety (25.9%), and hypertension (25.9%). Nearly all patients (94.4%) were using NSAIDs, while 48.1% were on gabapentinoids.

Physical examination revealed high rates of positive extension (87.0%) and rotation pain (88.9%), with paraspinal and segmental tenderness present in 48.1% and 37.0%, respectively. Radiologically, facet degeneration was mostly mild to moderate, and disc degeneration was predominantly mild (59.3%). PRF was most applied at three levels (51.9%), with C4–5 (77.8%), C3–4 (68.5%), and C5–6 (50.0%) being the most frequently treated segments.

### 3.3. Changes in VAS and NDI Scores over Time

Changes in VAS and NDI scores over time are shown in Table 2. Both VAS and NDI scores demonstrated statistically significant improvements over time (Friedman test: χ^2^ = 114.75 and χ^2^ = 57.97, respectively; both *p* < 0.001). The median VAS reduced from 7 at baseline to 4 at one month, with scores remaining at 5 at both three and six months. Median NDI decreased from 20 at baseline to 12 at 1 month, 14 at 3 months, and 16 at 6 months. Post hoc Bonferroni-corrected analyses demonstrated significant differences between baseline and all follow-up periods (all *p* < 0.001).

Anchor-based MCID values were calculated using the 13 patients who demonstrated 20–50% VAS improvement at 6 months as reference, yielding 2.23 points for VAS and 1.31 points for NDI.

### 3.4. Six-Month Treatment Outcome and Associated Factors

Treatment success, defined as ≥50% reduction in VAS score at 6 months, was achieved in 19 of 54 patients (35.2%). Comparison of demographic, clinical, and radiological characteristics between successful and unsuccessful groups is presented in Table 3.

No significant differences were found between groups regarding demographic characteristics including age (*p* = 0.292), sex (*p* = 0.263), and BMI (*p* = 0.978); however, occupational distribution was statistically significantly different (*p* < 0.001). Notably, all patients in the successful group were engaged in light (57.9%, n = 11) or medium (42.1%, n = 8) work, while none of the 14 patients with heavy work and none of the 4 sedentary workers were in the successful group.

Evaluation of clinical characteristics revealed significantly shorter pain duration in the successful group (median: 12 months vs. 24 months; *p* = 0.015). Success rates were significantly higher in patients with bilateral pain localization (47.4% vs. 8.6%; *p* = 0.003). Gabapentinoid use was significantly higher in the unsuccessful group (62.9% vs. 21.1%; *p* = 0.004). Among physical examination findings, paraspinal tenderness (84.2% vs. 28.6%; *p* < 0.001) and segmental tenderness (68.4% vs. 20.0%; *p* < 0.001) were markedly more frequently positive in the successful group.

Regarding PRF treatment levels, C3–4 level treatment was more common in the unsuccessful group (80.0% vs. 47.4%; *p* = 0.030), while C6–7 level treatment was more frequent in the successful group (52.6% vs. 22.9%; *p* = 0.037). A marked difference was observed between groups regarding medication dose changes (*p* < 0.001): medication doses could be reduced to 78.9% (n = 15) of successful group patients, compared to only 8.6% (n = 3) in the unsuccessful group.

Comparison of VAS and NDI scores between groups is presented in Table 4. Baseline VAS and NDI values were found to be significantly lower in the successful group (*p* < 0.001 for both). In the successful group, baseline median VAS score was 7 (IQR: 6–7), and median NDI score was 16 (IQR: 14.5–17.5), while in the unsuccessful group these values were 8 (IQR: 7–8) and 22 (IQR: 19.5–24.5), respectively. Significant differences between groups persisted at all follow-up periods for both absolute scores and percentage changes.

### 3.5. Predictive Factors for Six-Month Treatment Success

The outcomes of the logistic regression analysis are presented in Table 5. In the univariate analysis, eight variables demonstrated statistical significance (*p* < 0.05): pain duration, gabapentinoid use, paraspinal tenderness, segmental tenderness, PRF at C3–4 and C6–7 levels, baseline VAS, and baseline NDI scores.

**Figure 3:**
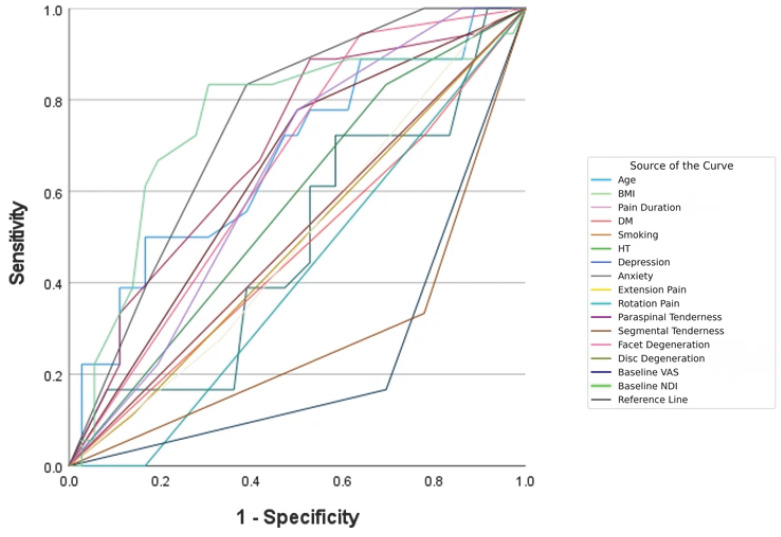
Receiver operating characteristic (ROC) curves for predictors of treatment success (≥50% VAS reduction at 6 months). Paraspinal tenderness (AUC = 0.764) and segmental tenderness (AUC = 0.722) demonstrated highest discriminative ability. Variables with AUC < 0.5 indicate inverse relationships where lower values predict favorable outcomes. Abbreviations: AUC, area under the curve; BMI, body mass index; DM, diabetes mellitus; HT, hypertension; NDI, Neck Disability Index; VAS, Visual Analog Scale.

Multivariate logistic regression using the Backward Wald method identified four independent predictors of treatment success: paraspinal tenderness (OR = 21.028; 95% CI: 1.845–239.689; *p* = 0.014), pain duration (OR = 0.923; 95% CI: 0.860–0.990; *p* = 0.026), baseline VAS (OR = 0.207; 95% CI: 0.054–0.785; *p* = 0.021), and baseline NDI (OR = 0.773; 95% CI: 0.616–0.969; *p* = 0.025). The discriminative ability of individual predictors is illustrated in Figure 3. The presence of paraspinal tenderness was strongly associated with treatment success (OR = 21.028; 95% CI: 1.845–239.689), while each additional month of pain duration, each one-point increase in baseline VAS, and each one-point increase in baseline NDI decreased success probability by 7.7%, 79.3%, and 22.7%, respectively.

## 4. Discussion

This study assessed the factors affecting the outcomes of ultrasound-guided CMB PRF treatment in patients experiencing chronic cervical facet joint pain. Our results indicated enhancements in pain intensity and functional disability after PRF treatment, achieving a treatment success rate of 35.2% at the 6-month mark. Multivariate analysis identified four independent predictors of treatment success: shorter pain duration, presence of paraspinal tenderness, lower baseline VAS, and lower baseline NDI. Among these, paraspinal tenderness emerged as the strongest predictor, substantially increasing treatment success probability, while longer pain duration was associated with decreased success. These findings underscore the importance of early intervention and careful patient selection based on clinical examination to optimize outcomes in CMB PRF.

In our study, treatment success defined as ≥50% VAS reduction at 6 months was achieved in 35.2% of patients, with VAS scores in the successful group decreasing from baseline (median 7) to 6 months (median 3), representing a 62.5% reduction. Similarly, NDI scores demonstrated a substantial improvement of 71.4% in the successful group. Yen et al., in their retrospective study of 204 patients evaluating PRF efficacy for cervical facet joint pain, reported a good outcome rate of 68.1% (139/204) [11]. The reduced success rate observed in our study can be related to variations in patient selection criteria, the use of a single diagnostic block protocol, and differences in the application of ultrasound-guided techniques. Jin et al. conducted a study assessing ultrasound-guided PRF to the C2 dorsal root ganglion for cervicogenic headache. They found that 68.97% of 29 patients experienced a ≥50% reduction in pain at the 24-week follow-up, with NDI scores decreasing from 35.21 ± 6.72 to 18.52 ± 8.04 [18]. Although the target structure differs, this study supports the reliability and efficacy of ultrasound-guided cervical PRF applications. In the randomized controlled trial by Alsaeid et al. comparing high-voltage PRF versus standard-voltage PRF for cervical radicular pain, the high-voltage group achieved a 100% success rate at 6 months (VAS: 86.5 to 20.4; NDI: 45.7 to 14.2), whereas no patient in the standard-voltage group achieved ≥50% VAS reduction during the same period (VAS: 86.1 to 80.3; NDI: 45.4 to 41.2) [19]. The technical parameters of radiofrequency treatment, including the rigor of diagnostic blocks, the number of lesions created, and the needle technique, are critically important determinants of clinical success. A systematic review and meta-analysis recently determined Level II evidence supporting the long-term effectiveness of cervical RF neurotomy for chronic facet-mediated neck pain management, noting significant variability in success rates according to procedural methodology [20]. The lower overall success rate in our study compared to some literature reports may be explained by patient demographic characteristics, pain duration, baseline severity, and occupational physical demands; notably, our multivariate analysis demonstrated that these factors independently influenced treatment success. Beyond patient-related factors, several methodological and mechanistic considerations may also contribute to the modest 35.2% success rate. PRF is a neuromodulatory rather than thermal neurolytic technique; Tekin et al. demonstrated that conventional RF produces more sustained pain relief through its heat lesion mechanism (80 °C), whereas PRF’s non-destructive effects at temperatures not exceeding 42 °C are characteristically shorter-lasting [21]. Additionally, the use of a single diagnostic block may have included false-positive responders, diluting the observed treatment effect. Finally, our strict ≥50% VAS reduction criterion at 6 months represents a stringent success threshold consistent with IMMPACT recommendations; less conservative criteria would yield higher reported success rates [17].

Our study revealed that paraspinal tenderness is the most significant independent predictor of treatment success, demonstrating a robust correlation with achieving a ≥50% reduction in pain at the 6-month mark. This association may be explained by the anatomical relationship between facet joints and paraspinal muscles. The CMBs provide innervation to the zygapophysial joints and the deep paraspinal muscles, specifically the multifidus and semispinalis cervicis. This finding aligns with the multi-center study conducted by Cohen et al., which assessed 92 patients undergoing cervical facet radiofrequency denervation and identified paraspinal tenderness as the sole clinical variable linked to treatment success [12]. While Engel et al. systematically found that success rates are distinctly lower when selection is based on clinical features alone, our data suggests that paraspinal tenderness may be a specific and valuable exception [22]. However, the extremely wide confidence interval (1.845–239.689) reflects substantial uncertainty in the point estimate due to small sample size and strong group separation, indicating a meaningful positive association rather than a precise effect magnitude. This finding supports its role as a key physical exam finding that can help identify patients most likely to benefit from subsequent, definitive diagnostic blocks as recommended. Regarding the reliability of paraspinal tenderness assessment, Schneider et al. reported substantial to excellent inter-rater reliability (κ = 0.74–0.96) for paraspinal tenderness in cervical facet joint pain patients using standardized palpation techniques [23]. In our study, a standardized assessment protocol was employed (firm digital pressure ~4 kg over C2–C7 facet joints, positive if reproducing characteristic pain or pain ≥4/10), which enhances clinical reproducibility. Nevertheless, formal inter-rater reliability assessment was not performed in this study, and future prospective studies should incorporate such evaluation.

Our multivariate analysis identified pain duration as an independent predictor of treatment success, with each additional month decreasing the probability of achieving ≥50% VAS reduction by 7.7% (OR = 0.923; *p* = 0.026). Patients in the successful group exhibited a significantly shorter median pain duration of 12 months compared to 24 months (*p* = 0.015). This finding is consistent with McCormick et al., who indicated that a shorter pain duration serves as a predictive factor for the clinical success of cooled radiofrequency ablation in patients experiencing pain from knee osteoarthritis [24]. The negative impact of prolonged pain duration may be attributed to central sensitization, characterized by increased excitability of central nervous system neurons and maladaptive changes in pain pathways. As chronic pain persists, neuromodulatory interventions targeting peripheral structures may have diminished efficacy once central sensitization is established. Interestingly, Cohen et al. did not find a significant relationship between pain duration and outcome in cervical facet radiofrequency denervation [12]. This discrepancy may reflect differences in patient populations, as their cohort had considerably longer pain durations overall. Our findings support early intervention in chronic cervical facet joint pain before extensive chronification develops.

An intriguing finding was that lower baseline VAS and NDI scores independently predicted treatment success. Patients with more severe pain and greater functional disability at baseline were less likely to achieve successful outcomes, suggesting that advanced disease states may be less responsive to PRF. Additionally, the relative ≥50% success threshold becomes harder to achieve at higher baseline values, and elevated pain scores may reflect established central sensitization or multi-source pathology beyond isolated facet joint origin. Van Boxem et al. similarly identified high disability as a negative predictor for PRF outcome in lumbosacral radicular pain [25]. Tseli et al., in their meta-analysis of 25 studies (n = 9436), confirmed that higher initial self-reported functioning predicted better outcomes after multidisciplinary rehabilitation [26]. Higher baseline scores may reflect advanced disease states with established central sensitization or psychosocial comorbidities that diminish the efficacy of peripheral interventions. These findings suggest that earlier intervention, before extensive functional deterioration occurs, may optimize treatment outcomes.

The finding that none of the patients engaged in heavy physical labor achieved treatment success is consistent with literature indicating poorer prognosis for neck pain recovery in blue-collar workers [27]. Although not retained as independent predictors in multivariate analysis, higher gabapentinoid use in the failure group suggests that pre-existing central sensitization may negatively impact PRF outcomes, as gabapentinoids primarily target centrally sensitized states; however, this observation is preliminary and highlights the need for further research to confirm the role of gabapentinoid use and central sensitization as prognostic factors. Similarly, the higher success rates at C6–7 compared to C3–4 may reflect the greater mechanical load and more defined nociceptive pathology at lower cervical segments, whereas upper cervical pain involves more complex referral patterns [28].

In our study, MCID values of 2.23 points for VAS and 1.31 points for NDI, which are relatively lower than previously reported values in cervical spine literature [29,30]. This discrepancy may be attributed to the fact that most MCID studies have been conducted following surgical interventions, which typically produce more dramatic changes; in contrast, minimally invasive techniques such as PRF may yield more modest yet clinically meaningful improvements in chronic facet joint pain populations.

This study has several strengths. First, all PRF procedures were performed under ultrasound guidance, which eliminates radiation exposure and allows real-time soft tissue visualization with Doppler assessment of vascular structures. Second, standardized patient selection criteria requiring ≥50% response to diagnostic medial branch block ensured population homogeneity. Third, comprehensive analysis of demographic, clinical, and procedural variables enabled identification of independent predictors. Finally, the six-month follow-up period represents a clinically meaningful timeframe for evaluating mid-term PRF efficacy.

This study has several limitations that warrant acknowledgment. The retrospective design inherently restricts the capacity to establish causal relationships and may introduce selection bias. Secondly, as a single-center study conducted at a tertiary pain clinic, the generalizability of our findings to other clinical settings may be restricted; multi-center studies would enhance the external validity and robustness of these results. Third, the sample size of 54 patients was determined by available cases rather than a priori power calculation. The multivariate model included four predictors with 19 events, yielding an events-per-variable ratio of 4.75, below the recommended threshold of 10. Post hoc analysis indicated 97% power for paraspinal tenderness, and the model exhibited excellent discrimination (AUC = 0.950) with satisfactory calibration (Hosmer–Lemeshow *p* = 0.965). However, the broad confidence interval for paraspinal tenderness (1.845–239.689) highlights the coefficient instability associated with small sample sizes. These findings are exploratory and necessitate validation in larger cohorts. Fourth, the use of a single diagnostic block rather than dual comparative blocks may contribute to a higher false-positive rate; however, this approach is supported by current consensus guidelines for both lumbar and cervical facet joint interventions, and has been employed in comparable multi-center research [12,28,]. Our strict ≥50% pain relief threshold partially mitigates this concern. Fifth, the 6-month follow-up period may inadequately reflect the long-term efficacy of the treatment; extending this duration could yield a more thorough understanding of the intervention’s sustained effectiveness. Sixth, while we noted the presence of depression and anxiety from patients’ medical histories, we did not utilize validated psychological assessment tools to quantify the severity of these conditions and their potential impact on patient-reported outcomes. As this is an observational study lacking a sham control group, the potential influence of placebo effects on the observed improvements cannot be ruled out. Future randomized controlled trials with larger sample sizes, extended follow-up periods, and controlled diagnostic blocks are necessary to validate these findings.

## 5. Conclusions

In this study, paraspinal tenderness was identified as the strongest positive predictor of ultrasound-guided CMB PRF treatment success, while longer pain duration, higher baseline VAS, and higher baseline NDI scores were associated with poorer outcomes. These findings suggest that early intervention in patients with localized facet joint pathology may improve PRF outcomes. The identified predictive factors can assist clinicians in patient selection and treatment expectations. Future randomized controlled trials with extended follow-up periods and research into optimal PRF parameters and predictive biomarkers are necessary.

## Figures and Tables

**Table 1:** Demographic, Clinical, and Radiological Characteristics of Patients.

Variables (N = 54)	n (%) or Median (IQR)
***Demographic Characteristics***	
Sex—Male/Female	23 (42.6)/31 (57.4)
Age (years)	54 (46–67)
BMI (kg/m^2^)	28.1 (25.9–31.3)
Occupation—Heavy/Medium/Light/Sedentary	14 (25.9)/23 (42.6)/13 (24.1)/4 (7.4)
***Clinical Characteristics***	
Pain duration (months)	21 (12–36)
Pain localization—Right/Left/Bilateral	23 (42.6)/19 (35.2)/12 (22.2)
Headache radiation	33 (61.1)
Shoulder radiation	15 (27.8)
History of trauma	11 (20.4)
Smoking/DM/Hypertension	22 (40.7)/13 (24.1)/14 (25.9)
Depression/Anxiety	6 (11.1)/14 (25.9)
NSAIDs/Opioids/Gabapentinoids	51 (94.4)/16 (29.6)/26 (48.1)
***Physical Examination Findings***	
Extension pain/Rotation pain	47 (87.0)/48 (88.9)
Paraspinal tenderness/Segmental tenderness	26 (48.1)/20 (37.0)
***Radiological Characteristics***	
Facet degeneration—Normal/Mild/Moderate/Severe	11 (20.4)/21 (38.9)/17 (31.5)/5 (9.3)
Disc degeneration—Normal/Mild/Moderate/Advanced	17 (31.5)/32 (59.3)/4 (7.4)/1 (1.9)
***PRF Procedure Characteristics***	
PRF Laterality—Right/Left/Bilateral	17 (31.5)/24 (44.4)/13 (24.1)
PRF levels—Two/Three/Four	22 (40.7)/28 (51.9)/4 (7.4)
PRF C2–3/C3–4/C4–5/C5–6/C6–7	21 (38.9)/37 (68.5)/42 (77.8)/27 (50.0)/18 (33.3)
Medication dose—Increased/Unchanged/Decreased	14 (25.9)/22 (40.7)/18 (33.3)

IQR: Interquartile range, BMI: Body mass index, DM: Diabetes mellitus, NSAIDs: Non-steroidal anti-inflammatory drugs, PRF: Pulsed radiofrequency. Occupational physical demands were classified according to the U.S. Bureau of Labor Statistics Occupational Requirements Survey (ORS) strength levels.

**Table 2:** Distribution of VAS and NDI Scores Over Time.

Variables (N = 54)	Median (IQR)	*p*-Value
**VAS scores**		
Baseline	7 (7–8)	<0.001 ᵃ
1 month	4 (3–5)	
3 months	5 (3–6)	
6 months	5 (3–7)	
**VAS change (%)**		
1 month	42.9 (25.9–57.1)	
3 months	35.4 (25.0–50.0)	
6 months	25.0 (14.3–55.4)	
**NDI scores**		
Baseline	20 (16–24)	<0.001 ᵃ
1 month	12 (7–18)	
3 months	14 (8–20)	
6 months	16 (5–24)	
**NDI change (%)**		
1 month	33.3 (10.3–56.9)	
3 months	24.0 (8.5–54.4)	
6 months	16.0 (0.0–61.3)	

ᵃ Friedman test. VAS: Visual analog scale, NDI: Neck disability index, IQR: Interquartile range. MCID (anchor-based): 2.23 points for VAS, 1.31 points for NDI.

**Table 3:** Comparison of Patients According to Six-Month Treatment Outcome.

Variables	Unsuccessful (n = 35)	Successful (n = 19)	*p*
***Demographics***			
Age (years)	54 (49–67)	54 (42–60)	0.292
Sex—Male	17 (48.6)	6 (31.6)	0.263
Occupation			<0.001
Heavy work	14 (40.0)	0 (0)	
Light work	2 (5.7)	11 (57.9)	
***Clinical***			
Pain duration (months)	24 (12–36)	12 (9–24)	0.015
Bilateral localization	3 (8.6)	9 (47.4)	0.003
Gabapentinoid use	22 (62.9)	4 (21.1)	0.004
***Physical Examination***			
Paraspinal tenderness	10 (28.6)	16 (84.2)	<0.001
Segmental tenderness	7 (20.0)	13 (68.4)	<0.001
***PRF Characteristics***			
PRF C3–4	28 (80.0)	9 (47.4)	0.030
PRF C6–7	8 (22.9)	10 (52.6)	0.037
Medication dose reduction	3 (8.6)	15 (78.9)	<0.001

Continuous variables are presented as median (IQR), categorical variables as n (%). Mann–Whitney U and Fisher’s exact/chi-square tests were used. Treatment success was defined as ≥50% VAS reduction at 6 months.

**Table 4:** Comparison of VAS and NDI Scores by Treatment Outcome.

Variables	Unsuccessful (n = 35)	Successful (n = 19)	*p*
**VAS scores**			
Baseline	8 (7–8)	7 (6–7)	<0.001
1 month	5 (3–6)	3 (3–4)	0.003
3 months	6 (4.5–6)	3 (3–3.5)	<0.001
6 months	6 (5–7)	3 (2–3)	<0.001
**VAS change (%)**			
1 month	37.5 (22.2–57.1)	50.0 (42.9–57.1)	0.029
3 months	28.6 (22.2–37.5)	57.1 (46.4–62.5)	<0.001
6 months	16.7 (12.5–25.0)	62.5 (53.6–69.0)	<0.001
**NDI scores**			
Baseline	22 (19.5–24.5)	16 (14.5–17.5)	<0.001
1 month	16 (11–20)	8 (5–12.5)	<0.001
3 months	20 (14.5–22)	6 (5–10)	<0.001
6 months	22 (17.5–25)	5 (4–5.5)	<0.001
**NDI change (%)**			
1 month	23.1 (4.9–54.4)	50.0 (32.6–61.2)	0.032
3 months	15.0 (4.5–23.1)	62.5 (48.7–66.7)	<0.001
6 months	0.0 (−8.5–15.4)	71.4 (60.2–75.0)	<0.001

Values are presented as median (IQR). Mann–Whitney U test was used. VAS: Visual analog scale, NDI: Neck disability index.

**Table 5:** Logistic Regression Analysis of Factors Predicting Six-Month Treatment Success.

Variable	Univariate OR (95% CI)	*p*	Multivariate OR (95% CI)	*p*
Age	0.965 (0.916–1.017)	0.187		
Pain duration (months)	0.942 (0.896–0.990)	0.019	0.923 (0.860–0.990)	0.026
Gabapentinoid use	0.158 (0.043–0.577)	0.005		
Paraspinal tenderness	13.333 (3.176–55.976)	<0.001	21.028 (1.845–239.689)	0.014
Segmental tenderness	8.667 (2.425–30.968)	0.001		
PRF C3–4	0.225 (0.066–0.765)	0.017		
PRF C6–7	3.750 (1.133–12.416)	0.030		
Baseline VAS	0.309 (0.148–0.646)	0.002	0.207 (0.054–0.785)	0.021
Baseline NDI	0.730 (0.609–0.876)	<0.001	0.773 (0.616–0.969)	0.025
*Model Diagnostics*				
Nagelkerke R^2^			0.731	
AUC (95% CI)			0.950 (0.883–0.989)	
Hosmer–Lemeshow χ^2^			1.41 (df = 6, *p* = 0.965)	
AIC/BIC			39.12/49.06	
*Classification Performance*				
Sensitivity/Specificity			78.9%/88.6%	
PPV/NPV			78.9%/88.6%	
Overall accuracy			85.2%	

Multivariate analysis was performed using the Backward Wald method. Variable selection was validated by comparing all 256 possible subsets of the 8 univariate-significant candidates using AIC and BIC; the final model ranked #3 by AIC (ΔAIC = 0.91 from the best model, indicating essentially equivalent fit) and #1 by BIC, confirming model parsimony. VIF values < 5 indicate acceptable multicollinearity. Baseline VAS and NDI showed moderate correlation (ρ = 0.45, *p* = 0.001) but retained independent predictive value. Classification metrics were calculated at the standard 0.5 probability cutoff. OR: Odds ratio; CI: Confidence interval; VIF: Variance inflation factor; AUC: Area under the receiver operating characteristic curve; AIC: Akaike Information Criterion; BIC: Bayesian Information Criterion; PPV: Positive predictive value; NPV: Negative predictive value; VAS: Visual analog scale; NDI: Neck disability index; PRF: Pulsed radiofrequency. Treatment success was defined as ≥50% VAS reduction at 6 months.

## Data Availability

The datasets generated and analyzed during the current study are not publicly available due to patient privacy considerations but are available from the corresponding author on reasonable request, subject to appropriate data sharing agreements and institutional approval.
